# Differential genetic responses to the stress revealed the mutation-order adaptive divergence between two sympatric ginger species

**DOI:** 10.1186/s12864-018-5081-3

**Published:** 2018-09-21

**Authors:** Bing-Hong Huang, Yuan-Chien Lin, Chih-Wei Huang, Hsin-Pei Lu, Min-Xin Luo, Pei-Chun Liao

**Affiliations:** 1 School of Life Science, National Taiwan Normal University, No. 88, Sec. 4, Ting-Chow Rd., Wenshan Dist, Taipei, 11677 Taiwan; 2 Department of Forestry, National Chung-Hsing University, No. 250, Kuo Kuang Rd, Taichung, 402 Taiwan

**Keywords:** Adaptive divergence, Divergent time, Ecological speciation, Mutation-order mechanism, Plastic divergence, Positively selected DEGs

## Abstract

**Background:**

Divergent genetic responses to the same environmental pressures may lead sympatric ecological speciation possible. Such speciation process possibly explains rapid sympatric speciation of island species. Two island endemic ginger species *Zingiber kawagoii* and *Z. shuanglongensis* was suggested to be independently originated from inland ancestors, but their island endemism and similar morphologies and habitats lead another hypothesis of *in situ* ecological speciation. For understanding when and how these two species diverged, intraspecific variation was estimated from three chloroplast DNA fragments (cpDNA) and interspecific genome-wide SNPs and expression differences after saline treatment were examined by transcriptomic analyses.

**Results:**

Extremely low intraspecific genetic variation was estimated by cpDNA sequences in both species: nucleotide diversity π = 0.00002 in *Z. kawagoii* and no nucleotide substitution but only indels found in *Z. shuanglongensis*. Nonsignificant inter-population genetic differentiation suggests homogenized genetic variation within species. Based on 53,683 SNPs from 13,842 polymorphic transcripts, in which 10,693 SNPs are fixed between species, *Z. kawagoii* and *Z. shuanglongensis* were estimated to be diverged since 218~ 238 thousand generations ago (complete divergence since 41.5~ 43.5 thousand generations ago). This time is more recent than the time of Taiwan Island formation. In addition, high proportion of differential expression genes (DEGs) is non-polymorphic or non-positively selected, suggesting key roles of plastic genetic divergence in broaden the selectability in incipient speciation. While some positive selected DEGs were mainly the biotic and abiotic stress-resistance genes, emphasizing the importance of adaptive divergence of stress-related genes in sympatric ecological speciation. Furthermore, the higher proportional expression of functional classes in *Z. kawagoii* than in *Z. shuanglongensis* explains the more widespread distribution of *Z. kawagoii* in Taiwan.

**Conclusions:**

Our results contradict the previous hypothesis of independent origination of these two island endemic ginger species from SE China and SW China. Adaptive divergent responses to the stress explain how these gingers maintain genetic differentiation in sympatry. However, the recent speciation and rapid expansion make extremely low intraspecific genetic variation in these two species. This study arise a more probable speciation hypothesis of sympatric speciation within an island via the mutation-order mechanism underlying the same environmental pressure.

**Electronic supplementary material:**

The online version of this article (10.1186/s12864-018-5081-3) contains supplementary material, which is available to authorized users.

## Background

Positive divergent selection is a key for driving or maintaining the species divergence in sympatry [1]. Particularly for the island species which usually physical contact, endogenous genetic barrier is important to the reproductive isolation between species of incipient speciation [2, 3]. In fact, ecological speciation could be much faster if selective pressures act on stress-related genes (e.g. cold resistance and starvation tolerance in *Drosophila melanogaster* [4]). Differential responses to environmental pressures reinforce and fasten the reproductive isolation [5–7]. If these differential responses have similar fitness advantages, selection may promote and maintain the incompatibility of genotypic combinations and is against gene flow via the Bateson-Dobzhansky–Muller incompatibility mechanism [8, 9]. This process particularly commonly used to explain the parapatric speciation of continental-island species with a continental relative [9]. In addition, ragged topography also creates ecological and altitudinal separation between populations/species (e.g. genera *Howea*, *Metrosideros* and *Coprosma* in Lord Howe Island [10–12]) that facilitates the speciation and radiation of island species. No matter parapatic or sympatric speciation of continental-island species, the natural selection usually involves in during speciation [13–16], despite a certain cases focused on the geographic isolation (allopatric speciation) [17–20].

**Fig. 1:**
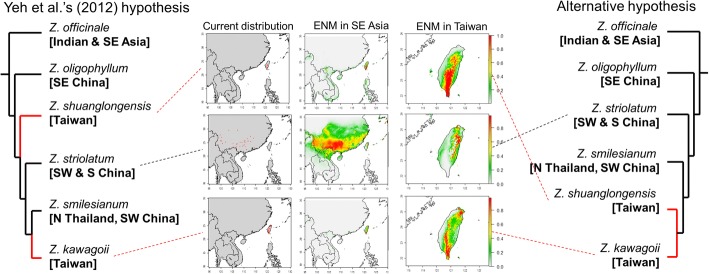
Phylogenetic hypotheses of *Z. kawagoii* and *Z. shuanglongensis* and allies and the distribution pattern of *Z. kawagoii*, *Z. shuanglongensis*, and *Z. striolatum*. The left plot shows the phylogenetic inference reconstructed by Yeh et al. [23]. The right panel is an alternative phylogenetic hypothesis inferred according to the distribution. Panels in the middle of figure reveal the current distribution, potential spatial distribution inferred by Ecological Niche Modeling (ENM) in the southeastern Asian and Taiwan. The current distribution is collected from GBIF database. It is worth to notify that *Z. striolatum* is not distributed in Taiwan, but the ENM suggests that the Taiwan Island is proper for its distribution. The current distribution and ENM of *Z. smilesianum* are not shown because of only one record in GBIF

Several novel species of genus *Zingiber* Mill. (Zingiberaceae Martinov) in Southeast Asia have been published due to distinguishable floral variation [21–24] while some ambiguous species were revised and combined [25] in recent years, implying some outstanding taxonomic problems in this genus in SE Asia. There are four native *Zingiber* species in Taiwan, a continental island off the continental Asia with a central mountain range up to near 4000-m height [26]. Among these four island natives, *Z. kawagoii* Hayata and *Z. shuanglongensis* C. L. Yeh & S. W. Chung are endemic to Taiwan and similar in morphology except slight differences of the rhizome color, bract size and color, and labellum color [26]. However, these two island endemic gingers were suggested to be phylogenetically separated by two inland allies *Z. smilesianum* Craib and *Z. striolatum* Diels that distributed in southwestern China (Fig. 1) [23].

*Zingiber oligophyllum*, previously reported as endemic to Taiwan, is now recorded for southeastern mainland China and Hong Kong [27]. Therefore, according to Yeh et al.’s [23] phylogenetic inference, *Z. kawagoii* and *Z. shuanglongensis* could be independently derived from widespread ancestors (Fig. 1). However, phylogenetic inference based on few genetic markers is easily affected by incomplete lineage sorting, particularly in species of frequent hybridization such as species of Zingiberaceae [28, 29]. In addition, *Z. smilesianum* and *Z. striolatum*, the sisters of *Z. kawagoii*, are widespread in SE Asia but not distributed in Taiwan. Therefore, we propose another hypothesis of recent and sympatric speciation for *Z. kawagoii* and *Z. shuanglongensis* according to their morphological similarity, island endemism and sympatric distribution (Fig. 1).

However, despite sympatric in Taiwan, some altitudinal differences between *Z. kawagoii* and *Z. shuanglongensis* are predicted by ecological niche modeling (ENM) based on 19 bio-climatic data from WorldClim database (Fig. 1). This suggests Grinnellian niche differentiation between these two species. According to different hypotheses of species origination, such niche differentiation could be a cause (e.g. sympatric ecological speciation) or a consequence (e.g. different originations or allopatric speciation) of speciation: if the former, we expected to detect genomic signatures of positive selection (i.e. adaptive divergence); if the latter, no or few response difference to the environmental pressures is expected.

In fact, the real distributional elevations of these two species are overlap (*Z. kawagoii*: 300–1500 m; *Z. shuanglongensis*: 1000–1400 m) with similar microhabitats (under forest, forest edge, slopes, and valley) [26]. This implies these two ginger species suffer similar environmental pressures. Therefore, if there is an adaptive divergence between *Z. kawagoii* and *Z. shuanglongensis*, it should be the mutation-order speciation [5, 30], says different mutations fixed in separate populations adapting to similar selective pressure leading divergence, rather than the divergent selection between different environments [5]. Exogenous stress is a major driver leading ecological divergence via selection [2]. Hence the genomic signature of adaptive divergence to the same stress was used for testing the mutation-order mechanism in this study.

Except genetic change, expressional plasticity plays another adaptive component to environmental change [31], by which different species with the same genotype may have differential expressions to response the same stress, i.e. reaction norms [32]. Many researches have indicated the key roles of plastic divergence for the incipient ecological speciation [31, 33, 34]. We tested two contrast hypotheses of adaptive vs. plasticity divergence between two ginger congeners. The genetic response to the stress only different in expression with no signals of positive selection (i.e. no nucleotide difference, selective constraints, and neutrality) is defined as plastic divergence. In contrast, the genes that are differentially expressed and positively selected are defined as adaptive divergence.

In this study, the genome-wide analysis is used to answer question about when and how *Z. kawagoii* and *Z. shuanglongensis* divergence. The objectives of this study were twofold: the first, to clarify whether these two island species are recently divergent in Taiwan or independently derived from inland ancestors; second, to test whether and how selective pressure drives or maintains the divergence in incipient speciation. To solve these questions, the chloroplast DNA (cpDNA) sequences were used to evaluate the intraspecific polymorphisms and interspecific divergence, and the genome-wide single nucleotide polymorphisms (SNPs) and RNA expression between these two species were collected via transcriptomic (RNA-seq) technology. Both sampled species were treated with saline before the experiment of RNA-seq in order to understand the genetic response under the stress of osmotic pressure change. Besides, such mild stress helps to increase overall transcription level and is conducive to obtain more loci for inferring the divergence of these two ginger congeners [35, 36]. The obtained SNPs were collected for divergent time estimation, and missense and silent SNPs were identified to detect signals of positive selection. Cross-reference of differential expression genes (DEGs) with positively selected genes (PSGs) was further used to determine the adaptive and/or plastic divergence between *Z. kawagoii* and *Z. shuanglongensis*. This study indicates a key role of genome-wide plastic divergence with adaptive divergence in stress-resistance genes between incipient species in sympatry.

## Results

### Low intraspecific genetic variation estimated by cpDNA in both species

We adopted three cpDNA region to evaluate the intraspecific divergence before inferring the genome-wide divergence between these two ginger congeners, including *trn*H-*psb*A intergenic spacer, *rpl*16 intron, and *trn*L-*trn*F intergenic spacer. Total nine *Z. kawagoii* populations and three *Z. shuanglongensis* populations were sampled (Table 1), which cover known distribution of these two species. The concatenated cpDNA sequences revealed very low intraspecific variation in these two species (nucleotide diversity π = 0.00002 in *Z. kawagoii*, while only indels but no SNP were found in *Z. shuanglongensis*). Furthermore, very low interpopulation divergences were observed in these two ginger congeners (Table 2, all corrected intraspecific π_XY_ < 0.001, all intraspecific *F*_ST_, the index of population differentiation, were nonsignificant). These results implied very little intraspecific variation, and the low intraspecific variation might be critical during inferring the divergence between species [37].

**Table 1:** Sampled population in chloroplast sequence amplification

population code	location	sample size	latitude	longitude
*Z. kawagoii*				
ZK_DSS	Dahsushan	8	N24.233	E120.900
ZK_WLS	Weiliaoshan	7	N22.879	E120.646
ZK_WLFS	Wulai, Fushan	9	N24.777	E121.504
ZK_RS	Rueishuei	9	N23.511	E121.330
ZK_NZ	Nanzhuang	10	N24.574	E121.044
ZK_KTS	Kantoushan	6	N23.267	E120.501
ZK_JIL	Jiajinlin	8	N22.402	E120.840
ZK_GK	Gukeng	10	N23.631	E120.617
ZK_DTDL	Datong Dali Trail	12	N24.192	E121.637
*Z. shuanglongensis*				
ZS_DLS	Dulanshan	12	N22.894	E121.186
ZS_EJT	Erjituan	10	N23.064	E120.717
ZS_LJ	Lijia Forest Trail	10	N22.805	E121.032

**Table 2:** Pairwise *F*_ST_ for population for population divergence in *Z. kawagoii* (ZK) and *Z. shuanglongensis*. The corrected average interpopulation difference (π_XY_) were shown above the diagonal, while the pairwise *F*_ST_ were shown below the diagonal

	ZK_DDS	ZK_WLS	ZK_WLFS	ZK_RS	ZK_NZ	ZK_KTS	ZK_JIL	ZK_GK	ZK_DTDL	ZS_DLS	ZS_EJT	ZS_LJ
ZK_DDS	–	0.000	0.000	0.000	0.000	0.000	0.000	0.000	0.000	22.171*	22.171*	22.171*
ZK_WLS	0.037	–	0.000	0.000	0.000	0.000	0.000	0.000	0.000	22.163*	22.163*	22.163*
ZK_WLFS	0.024	0.002	–	0.000	0.000	0.000	0.000	0.000	0.000	22.163*	22.163*	22.163*
ZK_RS	0.000	0.038	0.025	–	0.000	0.000	0.000	0.000	0.000	22.152*	22.152*	22.152*
ZK_NZ	0.000	0.069	0.059	0.000	–	0.000	0.000	0.000	0.000	22.161*	22.161*	22.161*
ZK_KTS	0.000	0.017	0.000	0.000	0.000	–	0.000	0.000	0.000	22.161*	22.161*	22.161*
ZK_JIL	0.000	0.026	0.011	0.000	0.000	0.000	–	0.000	0.000	22.171*	22.171*	22.171*
ZK_GK	0.000	0.026	0.011	0.000	0.000	0.000	0.000	–	0.000	22.16*	22.16*	22.16*
ZK_DTDL	0.000	0.051	0.040	0.000	0.000	0.000	0.000	0.000	–	22.161*	22.161*	22.161*
ZS_DLS	1.000*	0.996*	0.996*	1.000*	1.000*	1.000*	1.000*	1.000*	1.000*	–	0.000	0.000
ZS_EJT	1.000*	0.996*	0.996*	1.000*	1.000*	1.000*	1.000*	1.000*	1.000*	0.000	–	0.000
ZS_LJ	1.000*	0.996*	0.996*	1.000*	1.000*	1.000*	1.000*	1.000*	1.000*	0.000	0.000	–

*Significance of random allelic permutation (*P* < 0.05)

### Species divergence between two ginger congeners estimated from transcriptomes

A total of 14.59 and 14.70 million paired-end high-quality reads, in which 2.189 and 2.206 billion bases, with sequence error rate < 1% (Q20 percentages) of 96.36% and 96.68% were generated from transcriptomes of *Z. kawagoii* and *Z. shuanglongensis*, respectively. Totally 255,711 non-redundant transcripts with N50 value of 1378 bp were generated by Trinity *de novo* assembly. For pseudo-reference-based assembly, contigs of both *Z. kawagoii* and *Z. shuanglongensis* were assembled together by bowtie2 [38], which results in 205,168 reference sequences (129,806 and 138,497 transcripts of *Z. kawagoii* and *Z. shuanglongensis* aligned to pseudo-reference, respectively). The mean depth of coverage is 36.426 (±147.733, Additional file 1: Figure S1), and the mean mapping quality is 23.98.

To estimate species divergent time between *Z. kawagoii* and *Z. shuanglongensis*, 53,686 SNPs were identified in 13,842 (17,302,281 nucleotides) out of 205,168 transcripts (total length 137,601,681 nucleotides, average length 670.68 nucleotides per transcript), in which 91 transcripts are multi-allelic loci. The variant rate is 3.10 × 10^− 3^ per site. Among these SNPs, 10,693 SNPs are fixed differences (fixation rate is 6.18 × 10^− 4^ per site). If assuming a constant mutation rate, the divergent time *T* can be estimated by the formula *μ* = *K*/2*T*, where *μ* is the mutation rate and the *K* is the average variants per site or the average fixed differences per site. If applying the mutation rate 6.5 × 10^− 9^ or 7.1 × 10^− 9^ substitutions/site/generation estimated from *Arabidopsis thaliana* [39], the time to the most recent common ancestor (*TMRCA*) of both *Z. kawagoii* and *Z. shuanglongensis* could be coalesced back to roughly 2.38 × 10^5^~ 2.18 × 10^5^ generations ago when using variant rate 3.10 × 10^− 3^ per site as the *K*. If using the fixation rate (6.18 × 10^− 4^ per site) as *K* to estimate the divergent time, the species divergence was estimated to be 4.75 × 10^4^~ 4.35 × 10^4^ generations ago, which was suggested as the time of completion of speciation.

### The plastic species divergence inferred from high proportion of non-positively selected differential expression transcripts

**Fig. 2:**
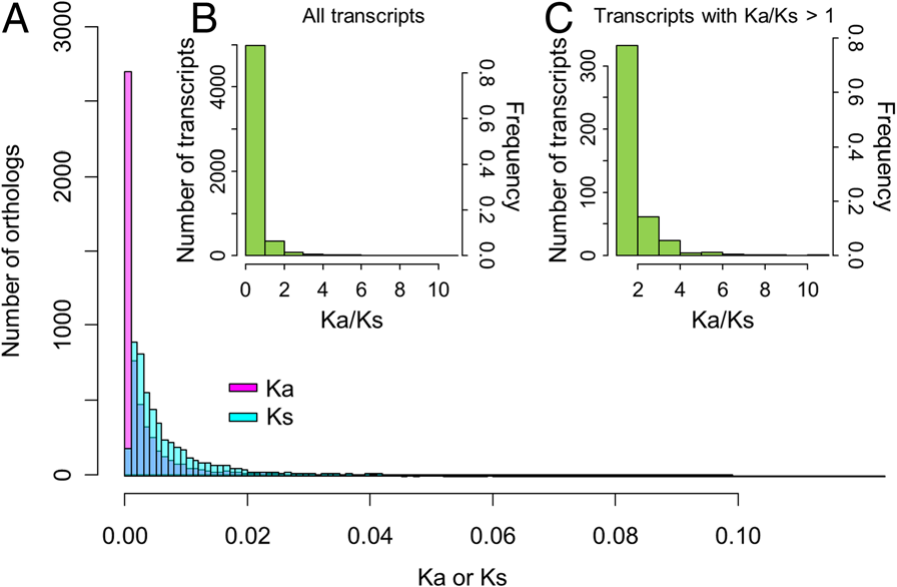
Distribution of the *Ka*, *Ks*, and *Ka*/*Ks* ratio of ortholog pairs under 60-min saline treatment. (**a**) Distribution of *Ka* and *Ks* values of all orthologous unigenes reveals a general patten of higher *Ks* than *Ka*; (**b**) L-shape *Ka*/*Ks* distribution in all transcripts revealed that most homologous genes (96.9% transcripts) of two ginger species are conserved (*Ka*/*Ks* < 1); (**c**) L-shape distribution of genes with *Ka*/*Ks* > 1 indicate that most of the positively selective pressures for the species divergence are slight (1 < *Ka*/*Ks* < 2)

Among these SNPs, 33,571 SNPs locate in exons (56.404%), 16,170 SNPs in intergenic regions (27.168%), 4 SNPs in the splice site region (0.007%), and 5466 (9.184%) and 4308 SNPs (7.238%) are found in the 5′ and 3′ untranslated region (UTR), respectively. Among 33,571 exonic SNPs, there are 14,967 missense variants (44.58%) and 18,393 synonymous variants (54.79%), and the other 213 SNPs are initiator codon variants, start lost, stop gained/lost, and stop retained variants (0.63%). Slightly smaller proportion of missense variants than the silent variants (44.58% vs. 54.79%) suggests that the overall genomic divergence of these two species is not far from neutral or nearly neutral evolutionary model [40]. The overall distributions of *Ka* and *Ks* values of orthologs are shown in Fig. 2.

**Fig. 3:**
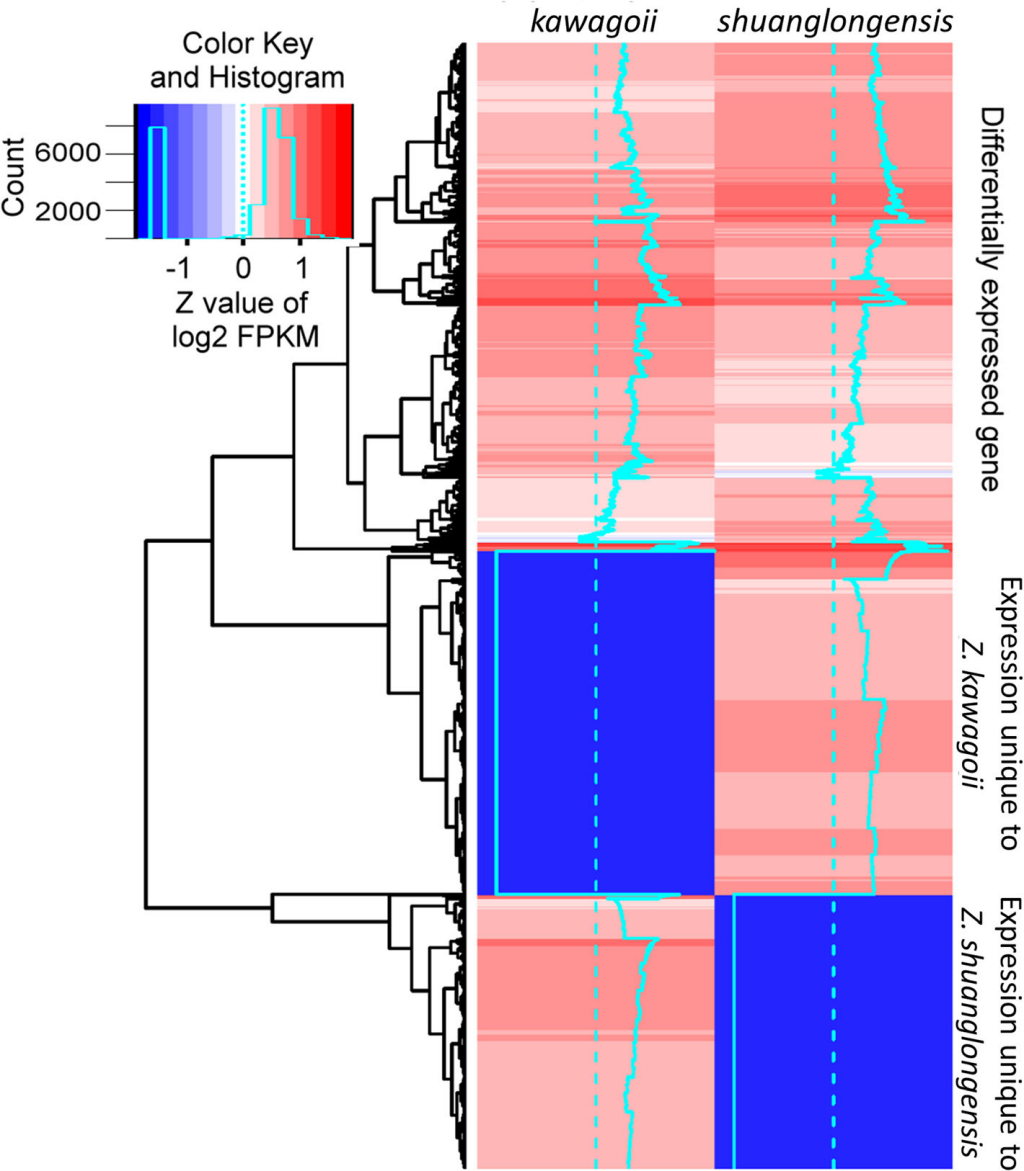
Comparison of relative expression (FPKM) of differentially expressed genes (DEGs) in heatmap. Only the DEGs fit criteria of log_2_FC > 1 and significant differences in FPKM (*P* < 0.05) are shown. Three major clusters of transcripts are the genes expressed in both species but differentially expressed and the genes expressed in single species only

We checked the differentially expressed genes using a criterion log_2_ fold-change (log_2_FC) > 1 and *P* < 0.05, resulting in 14,402 of 205,168 transcripts (7.02%) are differentially expressed. In these 14,402 DEGs, 3505 (24.34%) and 4396 transcripts (35.52%) are unique to *Z. shuanglongensis* and *Z. kawagoii*, respectively; while 6501 DEGs (45.14%) are commonly expressed in both species, in which 3176 (22.05%) and 3325 DEGs (23.09%) are higher expressed in *Z. shuanglongensis* and *Z. kawagoii*, respectively (Fig. 3). These differential expression patterns not only reflect the congenital differences in gene expression between species but also indicate divergent physiological responses to the saline stress.

**Fig. 4:**
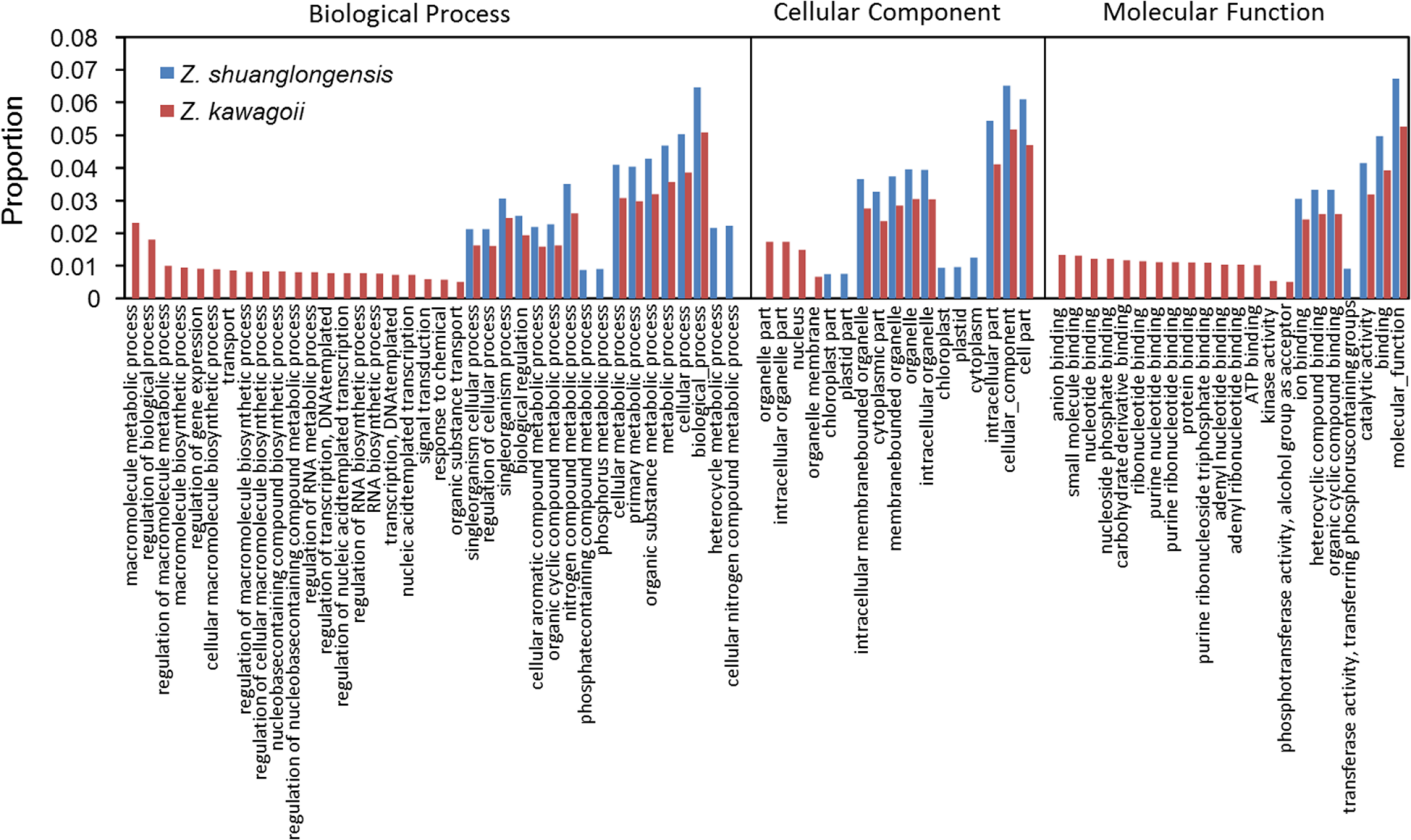
Differences of the gene ontology (GO) enrichments of three main categories between *Z. shuanglongensis* and *Z. kawagoii*. Only the GO terms with differences > 0.5% are shown. Apparently, more genes of certain GO terms are uniquely expressed in *Z. kawagoii* than in *Z. shuanglongensis*, particularly in categories Biological Process and Molecular Function

Based on the Gene Ontology (GO) annotation, 25,710 (50.00%), 14,296 (27.80%), and 11,184 (21.75%) unigenes of *Z. shuanglongensis* are identified as categories Biological Process (BP), Cell Component (CC), and Molecular Function (MF), respectively; 33,689 (50.00%), 15,295 (22.70%), and 28,473 (25.66%) unigenes are assigned as BP, CC, and MF, respectively, in *Z. kawagoii*. Comparisons of each functional class revealed higher proportion of unique expression of functional classes in *Z. kawagoii* than in *Z. shuanglongensis*, while the commonly expressed functional classes revealed higher expression level in *Z. shuanglongensis* than in *Z. kawagoii* (Fig. 4, the GO terms with differences > 0.5% are shown only). Such differences of expression patterns suggest that, in response to the stress, *Z. kawagoii* may quickly and widely trigger the expression of multiple functional genes (particularly the functional classes in BP and MF) in contrast to the concentrative expression of part of main functional categories in *Z. shuanglongensis*. The quick response and expression of diversified genes in the face of stresses may be advantageous to adapt changeable environment (i.e. adaptability) and increase the success of colonization (i.e. dispersability) [41, 42], which explains the broader habitat ranges of *Z. kawagoii* than *Z. shuanglongensis* in Taiwan (see the current distribution in Fig. 1).

**Fig. 5:**
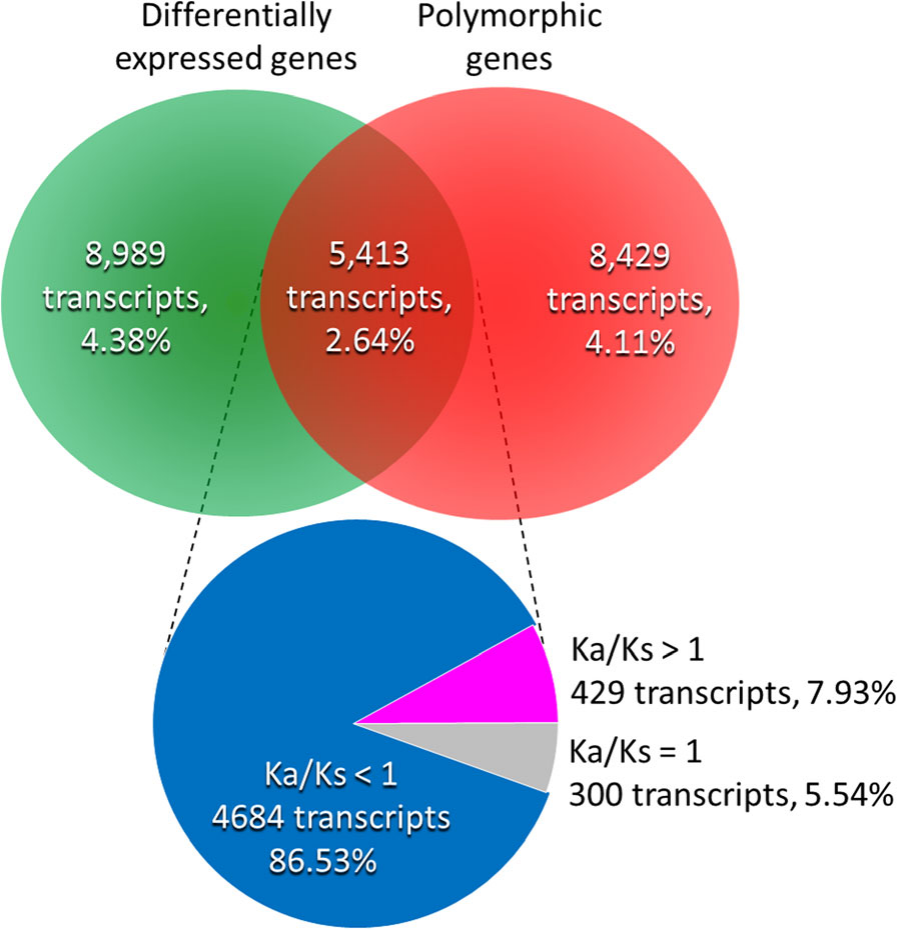
Venn diagram shows proportions of differentially expressed genes (DEGs) and polymorphic genes and the DEGs under positive selection (i.e. *Ka*/*Ks* > 1)

**Fig. 6:**
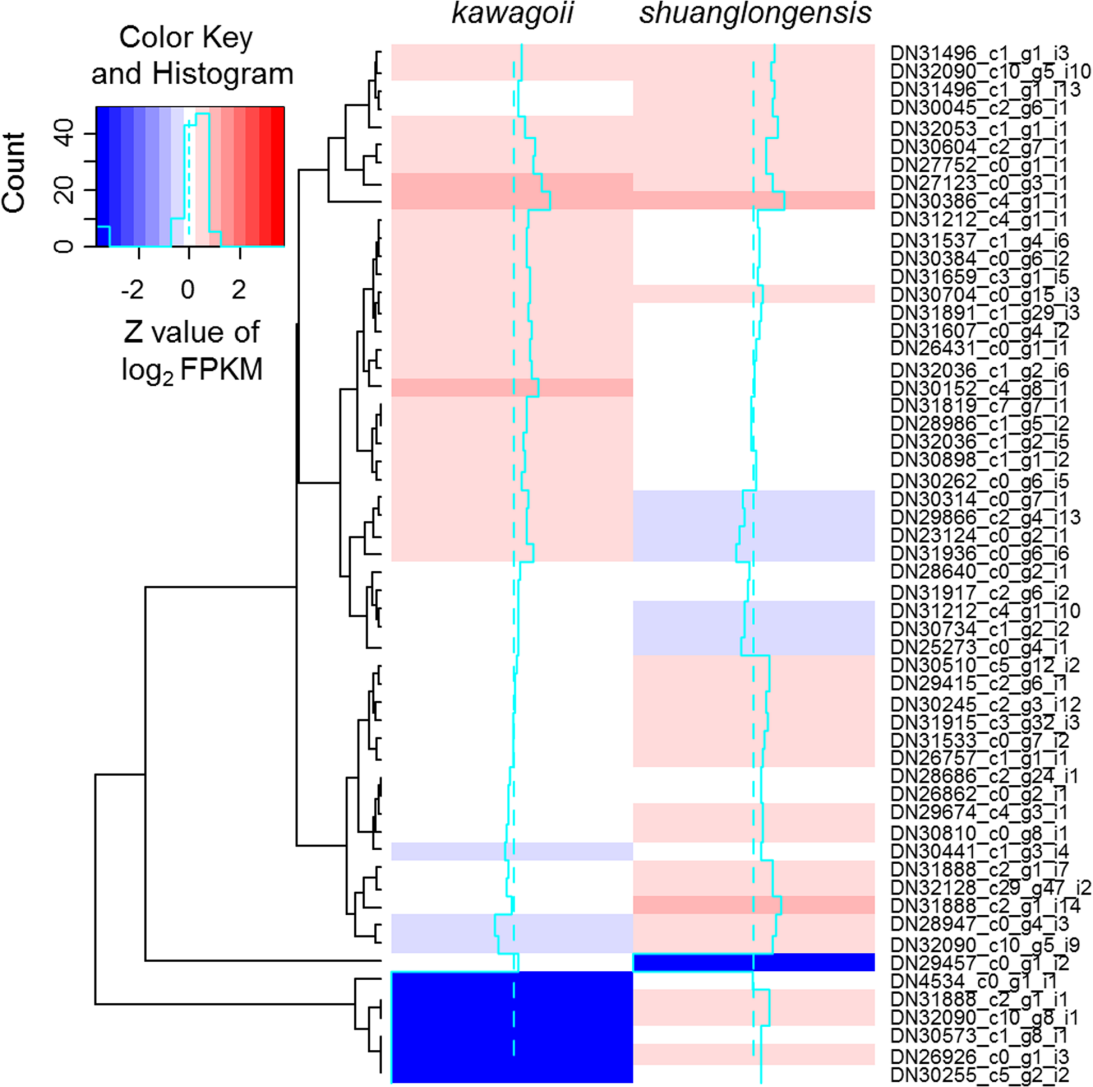
Comparison of FPKM of positively selected DEGs in heatmap. Only the DEGs fit criteria of *Ka*/*Ks* > 1, log_2_FC > 1 and significant differences in FPKM (*P* < 0.05) are shown

For testing whether such divergence is adaptive or just a phenomenon of plasticity, we further check the *Ka*/*Ks* ratio, a genetic signature of selection [43], of these DEGs. Among 5413 transcripts with exonic SNPs, small proportion of DEGs was found in transcripts with *Ka*/*Ks* > 1 (429/5413 = 7.93%, Fig. 5). In contrast, relatively high proportion of DEGs was negatively selected or under selective constraint (i.e. *Ka*/*Ks* < 1, 4684/5413 = 86.53%, Fig. 5). The result of high proportion of DEGs without under positive selection supports the plasticity hypothesis rather the adaptive hypothesis of genomic divergence between two ginger congeners. Within these positively selected DEGs inferred by log_2_FC > 1, only 56 DEGs were also significant differences in Fragment Per Kilobase of transcript per Million mapped reads (FPKM) (*P* < 0.05, Fig. 6), in which 15 DEGs were highly adaptive (*Ka*/*Ks* > 2, Table 3). These highly adaptive DEGs can be considered to be the key genetic agents that cause or maintain the differentiation of the two ginger congeners.

**Table 3:** List of SNPs and relative expressions of transcripts with *Ka*/*Ks* > 2 and significantly differential expression

		SNPs			FPKM			
Transcript Id	Length	Missense	Synonymous	*Ka*/*Ks*	*Z. kawagoii* (zk)	*Z. shuanglongensis* (zs)	log_2_FC (zs-zk)	*p* (zs-zk)
DN30045_c2_g6_i1	386	6	1	3	5.12	26.67	2.29	0.038
DN32090_c10_g8_i1	634	5	1	2.5	0	17.47	7.97	1.80E-05
DN32090_c10_g5_i9	722	5	1	2.5	0.44	27.29	5.67	1.09E-06
DN31496_c1_g1_i13	839	16	2	4	5.62	31.54	2.36	0.002
DN32036_c1_g2_i5	1148	13	2	3.3	9.84	1.85	−2.61	0.002
DN31537_c1_g4_i6	1778	12	2	3	14.86	4.92	−1.77	0.016
DN31659_c3_g1_i5	2061	6	1	3	20.03	4.67	−2.27	0.002
DN28640_c0_g2_i1	2844	6	1	3	5.84	1.72	−1.91	0.013
DN30152_c4_g8_i1	403	5	1	2.5	55.55	2.79	−4.29	1.13E-04
DN26431_c0_g1_i1	1066	6	1	3	22.23	3.39	−2.85	3.77E-04
DN30898_c1_g1_i2	1872	5	1	2.5	11.4	3.66	−1.8	0.015
DN32090_c10_g5_i10	1862	46	7	3.3	7.98	24.08	1.44	0.039
DN31607_c0_g4_i2	2162	11	2	2.8	27.88	5.09	−2.6	3.37E-04
DN31888_c2_g1_i14	465	7	1	3.5	2.15	83.59	5.16	5.25E-07
DN26862_c0_g2_i1	1481	7	1	3.5	1.62	6.52	1.85	0.032

### The adaptive divergence between species inferred from positive selection on stress-resistance DEGs

We further checked the functions of these highly adaptive-divergent (i.e. *Ka*/*Ks* > 2) DEGs. Ten of 15 transcripts were annotated (Table 4). Five of ten annotated transcripts are disease resistance genes analogous to RGA1 (DN30898_c1_g1_i2), RGA2 (DN32090_c10_g5_i9, DN31496_c1_g1_i13), RPM1 (DN32036_c1_g2_i5), and RPP13-like protein 1 (DN32090_c10_g5_i10); one is analogous to Mitogen-activated protein kinase kinase kinase 1 (MEKK1) (DN26862_c0_g2_i1), with a function of signaling pathway to modulate gene expressions responding to biotic (pathogen defense by negatively regulating innate immunity) and abiotic stresses (cold and salinity stress-mediated MAP kinase signaling cascade); one is analogous to Peroxiredoxin-2C (DN26431_c0_g1_i1), playing a role in cell protection against oxidative stress; and one is an analog of Retrovirus-related Pol polyprotein from transposon TNT 1–94 which might transcribe in the face of stresses (DN28640_c0_g2_i1). These eight transcripts are related to the resistance of biotic and abiotic stresses. Positive selection on these stress-resistance DEGs indicated differential genetic responses to the stress may play important roles in driving or maintaining species divergence.

**Table 4:** Functional annotation of 15 genes listed in Table 1

			BLASTP		BLASTX						
Transcript Id	UniprotKB gene	Protein	e value	bit score	e value	bit score	Pfam	COGs (EggNog)	Pathways	EC number	KEGGs
DN30045_c2_g6_i1	–		–	–	–	–	.	.	–	–	.
DN32090_c10_g8_i1	–		–	–	–	–	.	.	–	–	.
DN32090_c10_g5_i9	RGA2_SOLBU	Disease resistance protein RGA2	1.80E-23	101	3.16E-24	104	PF00931.21: NB-ARC domain	.	–	–	.
DN31496_c1_g1_i13	RGA2_SOLBU	Disease resistance protein RGA2	4.85E-13	71.6	2.33E-09	61.6	PF00931.21: NB-ARC domain	.	–	–	.
DN32036_c1_g2_i5	RPM1_ARATH	Disease resistance protein RPM1	3.33E-48	176	1.61E-52	191	.	COG4886: leucine Rich Repeat	osa04626 (Plant-Pathogen Interaction)	–	KO:K13457
DN31537_c1_g4_i6	WAK2_ARATH	Wall-associated receptor kinase 2	3.10E-133	408	2.03E-132	407	PF13947.5: Wall-associated receptor kinase galacturonan-binding	COG0515: Serine Threonine protein kinase	–	2.7.11	.
							PF08488.10: Wall-associated kinase PF07645.14: Calcium-binding EGF domain				
							PF00069.24: Protein kinase domain				
							PF07714.16: Protein tyrosine kinase				
DN31659_c3_g1_i5	–	–	–	–	–	–	PF03732.16: Retrotransposon gag protein	.	–	–	.
DN28640_c0_g2_i1	POLX_TOBAC	Retrovirus-related Pol polyprotein from transposon TNT 1–94	1.52E-76	277	5.95E-62	233	PF14223.5: gag-polypeptide of LTR copia-type	.	–	2.7.7.49	.
							PF13917.5: Zinc knuckle			3.4.23	
							PF13976.5: GAG-pre-integrase domain				
							PF00665.25: Integrase core domain				
DN30152_c4_g8_i1	–		–	–	–	–	.	.	–	–	.
DN26431_c0_g1_i1	PRX2C_ARATH	Peroxiredoxin-2C	1.81E-88	264	4.42E-85	257	PF02966.15: Mitosis protein DIM1	COG0678: peroxiredoxin	–	1.11.1.15	.
							PF08534.9: Redoxin			1.11.1.7	
							PF00578.20: AhpC/TSA family				
DN30898_c1_g1_i2	RGA1_SOLBU	Putative disease resistance protein RGA1	3.29E-34	141	1.14E-33	141	.	.	–	–	.
DN32090_c10_g5_i10	R13L1_ARATH	Putative disease resistance RPP13-like protein 1	2.70E-41	164	2.45E-31	134	PF13855.5: Leucine rich repeat	COG4886: leucine Rich Repeat	–	–	.
							PF13516.5: Leucine Rich repeat				
DN31607_c0_g4_i2	–		–	–	–	–	.		–	–	.
DN31888_c2_g1_i14	C71A1_ZINZE	Alpha-humulene 10-hydroxylase	3.00E-90	274	3.34E-90	274	PF00067.21: Cytochrome P450		–	1.14.13.150	.
DN26862_c0_g2_i1	M3K1_ARATH	Mitogen-activated protein kinase kinase kinase 1	9.38E-72	242	3.62E-60	211	PF00069.24: Protein kinase domain	ENOG410XQGS: mitogen-activated	osa04016 (Plant-Pathogen Interaction)	2.7.1	KO:K13414
							PF07714.16: Protein tyrosine kinase	protein kinase kinase kinase	osa04626 (MEPK Signaling Pathway)	2.7.11.25	

In addition to the transcript DN28640_c0_g2_i1 analogous to Retrovirus-related Pol polyprotein from transposon TNT 1–94, DN31659_c3_g1_i5 was also annotated as encoding a Retrotransposon gag protein (Table 4). Transcription of TE is known to increase under various kinds of stresses in several systems [44–46]. For example, in *Arabidopsis* [47] and coral *Acropora hyacinthus* [48], transposons and retrotransposons are activated during heat stress and are regulated by small interfering RNAs (siRNAs); *Copia* retrotransposons of the *Cucumis melo* genome are transcriptionally induced when melon leaves are exposed to UV light [49]; differentially expression of multiple retrovirus-related Pol polyproteins between two bryophytes with divergent desiccation tolerance during cycles of rapid desiccation and rehydration in the intertidal zone differentiates their ecological niches [50]. These two transposons and retrotransposons expressed higher in *Z. kawagoii* than in *Z. shuanglongensis* (Table 4). Another abiotic stress resistance transcript DN26431_c0_g1_i1 (analog of Peroxiredoxin-2C) is also higher expressed in *Z. kawagoii* (Table 4). The high expression of abiotic resistance genes in *Z. kawagoii* may be beneficial to the adaptation to a variety of environments, which explains the wider distribution of *Z. kawagoii* than *Z. shuanglongensis* in Taiwan.

The other two transcripts are analogous to the Alpha-humulene 10-hydroxylase (DN31888_c2_g1_i14) which is a catalytic enzyme of zerumbone biosynthesis with an anticancer function, and the Wall-associated receptor kinase 2 (DN31537_c1_g4_i6) functioning in the control of cell expansion, morphogenesis and development (Table 4). The positive selection and differential expression on these genes suggest differential morphogenesis and physiological functions between *Z. kawagoii* and *Z. shuanglongensis*. Ginger (*Z. officinalis* Rosc.) and other zingiberaceous plants have potent antioxidant and anti-inflammatory activities and are therefore suggested to exhibit cancer preventive activity [51–53]. Differential expression and positive selection on *Alpha-humulene 10-hydroxylase* analog (DN31888_c2_g1_i14) suggest that *Z. shuanglongensis*, which has higher expression level, has a higher anti-inflammatory or anticancer effect than *Z. kawagoii*, although more researches are necessary.

## Discussion

### The species divergence event was later than the island formation time

The divergence of these two ginger congeners was since 2.38 × 10^5^~ 2.18 × 10^5^ generations ago and completed at 4.75 × 10^4^~ 4.35 × 10^4^ generations ago. Since the variant rate could be overestimated due to post-speciation polymorphism, the real *TMRCA* may be even smaller than the estimation. Therefore, the divergence of these two island-endemic species is more recent than the time of island formation (< 5 million years ago (Mya) [54–56]). If Yeh et al.’s [23] hypothesis is true (Fig. 1), *Z. shuanglongensis*, *Z. striolatum*, *Z. smilesianum* and *Z. kawagoii* must be rapidly speciated and dispersed to Taiwan and SW China several times within hundreds of thousands of years (Fig. 1). Although this hypothesis cannot be completely exclusive due to lack of comparative genetic data of *Z. striolatum* and *Z. smilesianum*, multiple long-distance dispersal with speciation events (e.g. founder speciation) in these low-migratory plants is not easy in a short time. Alternatively, the hypothesis of *in situ* speciation within Taiwan Island (Fig. 1, the alternative hypothesis) is more probable to explain the sympatric and recent divergence between *Z. kawagoii* and *Z. shuanglongensis*. Anyhow, *Z. kawagoii* and *Z. shuanglongensis* are undisputedly incipient species.

### Adaptive plasticity broadens selectabilities in the incipient speciation

Differentiation of physiological responses to the stress can arise through phenotypic plasticity, divergent selection, or a combination thereof, leading to speciation [33, 57]. High proportion of plastic divergence (i.e. DEGs without signatures of positive selection) suggests rapid and pluralistic responses to environmental stresses (e.g. osmotic pressure change due to saline or drought) [58], which increases the variability and selectivity at the beginning of speciation [31]. Adaptive plasticity also facilitates the success of organism to colonize to new habitats, that potentially contributes to genetic differentiation and speciation [59]. The recent divergence between *Z. shuanglongensis* and *Z. kawagoii* since Middle-to-Late Pleistocene suggests that the speciation initiates in an era of rapid change of climate and environment. High proportion of plastic divergent DEGs facilitates the survival of the common ancestors of *Z. shuanglongensis* and *Z. kawagoii*. If the divergent genetic responses are equally advantageous to the environmental pressure, these two incipient species can persist [8]. Such plasticity extends the persistence of the tolerance of environmental change of populations in low genetic variability, which is common in island endemic species, and the following adaptive evolution can facilitate the persistent responses to environmental change [58, 60].

Certain nonsynonymous polymorphisms of DEGs are found to be fixed between *Z. shuanglongensis* and *Z. kawagoii*. Such divergent process is particularly revealed in pathogenic and stress-resistance genes (Table 4). In the study of *Brassica rapa*, pathogenic sustainability changes accompanying with the evolutionary shift of the drought response and even results in early flowering following the drought stress [61]. Although no obvious difference in flowering time between *Z. shuanglongensis* and *Z. kawagoii*, *Z. kawagoii* have unique expression of genes regarding the regulation of red or far-red light signaling pathway (GO:0090227). Within this GO term, the transcript DN28195_c2_g11_i1 (analog to the *REPRESSOR OF UV-B PHOTOMORPHOGENESIS 2* of *Arabidopsis thaliana*) that involves in the regulation of photoperiodic flowering and vegetative development have significant higher expression in *Z. kawagoii* than in *Z. shuanglongensis* (log_2_FC = 1.866, *P* = 0.009), and the transcript DN29957_c0_g6_i13 (analog to the Myb transcription factor *EARLY FLOWERING MYB PROTEIN*, of *A. thaliana*) that acts as a flowering repressor is positively divergent between *Z. shuanglongensis* and *Z. kawagoii* (*Ka*/*Ks* = 1.5). Although lack direct evidences to link the expressional differences regarding the pathogenic resistance genes with the flowering regulation under the drought stress, this transcriptomic data revealed clues that the expressional plasticity of stress- and pathogen-resistance genes might be associated with the evolution of a reproductive barrier toward adaptive divergence and ecological speciation [33].

### Positive selection on stress-resistance genes as an important rule underlying the maintenance of species divergence

Higher expression of genes related to responses to stress and pathogens reveals higher adaptability in *Z. kawagoii* than in *Z. shuanglongensis*. Studies on wild sunflower have shown that the positive selection for salt tolerance quantitative trait loci (QTLs) is strong enough to counteract the homogenizing effect of gene flow and facilitate the sympatric speciation [62, 63]. Divergent responses to the stresses (e.g. saline, pathogens, etc.) accompanying with the gamete incompatibility that results in reproductive barrier during incipient speciation is probably a pleiotropic effect of the evolution of stress- or pathogen-resistance genes, and the pleiotropic effect may lead to gamete defect and further support the scenario of Bateson-Dobzhansky-Muller incompatibility [3, 8, 9]. The island endemic with overlapping distributions of recently divergent *Z. kawagoii* and *Z. shuanglongensis* implies the sympatric speciation. Although the speciation drivers and speciation genes are unknown yet, the transcriptomic data revealed that positively divergent selection on stress- and pathogen-resistance DEGs is involved in blocking the genetic homogenization of gene flow between species. These stress- and pathogen-resistance DEGs are undoubtedly one of the important factors to maintain reproductive isolation in the incipient speciation of *Z. kawagoii* and *Z. shuanglongensis* in Taiwan. This is congruent with Bomblies and Weigel’s [3] autoimmunity hypothesis for the between-species incompatibility, and the adaptive divergent responses to the same stress support the mutation-order speciation sympatrically within an island.

## Conclusions

In conclusion, *Z. kawagoii* and *Z. shuanglongensis* is recently diverged and is most probably sympatrically speciated underlying the adaptive divergence of stress-resistance DEGs. Since lack of obvious habitat heterogeneity between species, we suggested mutation-order speciation, a mechanism of fixation of equally advantageous mutations in different populations/incipient species in the face of the same environmental pressures, might explain the adaptive divergence of these two sympatric island species. These adaptive-divergence mechanisms are based on the expressional plasticity of incipient speciation to expand adaptabilities of incipient species. Although the speciation mechanism and origination hypothesis need more studies to validate, differential responses to the same stress in genetic variation and expression have explained how sympatric incipient species diverged when suffering identical environmental pressures.

## Methods

### Plant material preparation, RNA extraction, and transcriptome sequencing

Mature plants of *Z. kawagoii* and *Z. shuanglongensis* are domesticated for more than a month in the greenhouse at the National Taiwan Normal University after collection from Miaoli Nanzhuang (N24°34′24.1″, E121°02′31.7″) and Jenlun Forest Road (N25°02′0.6″, E121°33′50.8″). Before RNA extraction, the roots of samples were irrigated with 1 M NaCl for saline stress for 1 h, and then the fresh leaves were collected for RNA extraction immediately using an improved cetyltrimethylammonium bromide (CTAB) method. RNA integrity was checked through 1% agarose gel electrophoresis, and then the mRNA was purified using poly-T oligo-attached beads. The cDNA libraries of both species were constructed from qualified RNA following manufacture’s protocol (Illumina Inc. San Diego, CA, USA). Library quality was validated on Agilent 2100 Bio-analyzer (Agilent Technology, CA, USA) and real-time PCR system. The paired-end sequencing was then performed by Illumina HiSeq platform. To elucidate the within-species variation of both Z. *kawagoii* and *Z. shuanglongensis*, we adopted three chloroplast regions for studying the level of intra-specific variation, including *trn*H-*psb*A intergenic spacer (forward primer: 5′-GTT ATG CAT GAA CGT AAT GCT C-3′, reverse primer: 5’-CGC GCA TGG TGG ATT CAC AAT C-3′), *rpl*16 intron (forward primer: 5’-CGA AAT CGG TAG ACG CTA CG-3′, reverse primer: 5′- ATT TGA ACT GGT GAC ACG AG-3′), and *trn*L-*trn*F intergenic spacer (forward primer: 5′- GCT ATG CTT AGT GTG TGA CTC GTT A-3′,reverse primer: 5’-CTT CCT CTA TGT TGT TTA CG-3′). We sampled nine *Z. kawagoii* populations and three *Z. shuanglongensis* populations (Table 1) for amplification and sequencing using BigDye terminator kit (Applied Biosystems). The divergence index including *F*_ST_ and average pairwise π_XY_ were adopted to elucidate the intraspecific diversity. The concatenated sequences were deposited on GenBank (accession number: MH758531-MH758541), and the aligned file was also deposited in Mendeley (https://data.mendeley.com/datasets/fky8hszf8n/1).

### Bioinformatics analysis

The modular tool MultiQC [64] was used for evaluating read quality. With the FastQC for quality control, the reads contaminated with adapter sequences, containing N bases accounting for > 5% of the total read length, or low-quality sequences with Phred score < 28 were removed using Trimmomatic [65]. Trinity ver. 2.3.2 [66] was used to *de novo* assembly with a setting of minimum contig length 150 bps and the CD-HIT-EST [67] was used for removing redundant transcripts to obtain more specific unigenes with a 95% sequence identity threshold. The filtered reads data were deposited in the NCBI Sequence Read Archive (SRA) with the Bioproject accession number PRJNA437070. The backbone reference was *de-novo* assembled using reads from both species by bowtie2 ver. 2.3.1 [38] and the read count quantification was performed by RSEM ver. 1.2.31 [68]. Relative expression level was calculated by the FPKM using RSEM ver. 1.2.31 [68]. Differential gene expression was then estimated by edgeR ver. 3.5 [69] with a criterion of log_2_FC > 1 with *P* < 0.05. We cross-compared the significant differences of FPKM (*P* < 0.05) and log_2_FC > 1 to confirm the differential expression.

We used the Transdecoder ver. 3.0.1 [https://transdecoder.github.io/] to parse coding region of all transcripts and retrieve protein sequences. Trinotate ver. 3.0.2 [https://trinotate.github.io/] was further used for functional annotation to processing the blastx and blastp homology search in UniProtKB/Swiss-Prot database. We also used the protein families (Pfam) domain database to identity protein domain in predicted and annotated proteins. Clusters of Orthologous Groups (COGs), Gene Ontology (GO), Kyoto Encyclopedia of Genes and Genomes (KEGG) functional and pathway annotation was also conducted. The GO enrichment analysis for gene set was further performed to compare the differences of functional expression groups between two species.

SNPs of homologous transcripts in both species were identified by samtools [70] and bcftools [71]. The SNPs data were saved as the Variant Call Format (VCF) file in which only the SNPs with quality > 30 were retained. The snpEff [72] and SnpSift [73] were used for SNP annotation and retrieve useful annotated variants, respectively. In the snpEff analysis, functional classes (missense, nonsense, and synonymous variants) and variant regions (3′- and 5’-UTR, intergenic, exonic, and splice-site regions) were classified.

### Testing the adaptive-divergence and plastic-divergence hypotheses

After the SNP calling, the ratio of missense mutation rate to silent mutation rate of each gene (hereafter named “*Ka*/*Ks*”) was estimated by assuming two fold nonsynonymous sites more than synonymous sites of coding sequences. Although this is a rough estimation, the estimated *Ka*/*Ks* ratio is better than direct estimating the proportion of nonsynonymous and synonymous variants (i.e. *pa*/*ps*) for the inference of positive selection (*Ka*/*Ks > 1*). Among all transcripts with exonic SNPs, we compared the proportion of DEGs to the number of no-expressional-difference genes between those of *Ka*/*Ks* > 1 and *Ka*/*Ks* ≤ 1. Since the differential gene expression between species indicates divergent physiological responses to the environment, the positive selection on DEGs indicates the adaptive divergence. In contrast, the DEGs with *Ka*/*Ks* ≤ 1 indicate the expressional divergence is not genetically based but an acclimated response, i.e. plastic divergence.

## Additional files

Additional file 1: Sequencing depth of coverage across pseudo-reference genome. (DOCX 653 kb)

## Abbreviations

BP: Biological Process of the GO term
CC: Cell Component of the GO term
COG: Clusters of Orthologous Groups
cpDNA: Chloroplast DNA
CTAB: Cetyltrimethylammonium bromide
DEG: Differential expression genes
ENM: Ecological niche modeling
FPKM: The Fragment Per Kilobase of transcript per Million mapped reads
*F*_ST_: The fixation index, a measurement of population differentiation
GO: Gene Ontology
*K*: The average variants (or fixed differences) per site
*Ka*: Nonsynonymous mutation rate
KEGG: Kyoto encyclopedia of genes and genomes
*Ks*: Synonymous mutation rate
log_2_FC: log_2_ fold-change
MF: Molecular Function of the GO term
Mya: Million years ago
Pfam: The protein families
PSGs: Positively selected genes
Q20: The sequencing quality score of 20 (an error rate of 1 in 100, with a corresponding call accuracy of 99%)
QTLs: Quantitative trait loci
RNA-seq: RNA sequencing or the transcriptome sequencing
siRNA: small interfering RNAs
SNPs: single nucleotide polymorphisms
SRA: Sequence Read Archive
*T*: The divergent time
*TMRCA*: The time to the most recent common ancestor
UTR: Untranslated region
VCF: The Variant Call Format
*μ*: Mutation rate
π: Nucleotide diversity estimated by pairwise differences
π_XY_: The average nucleotide diversity estimated by π
