# Supplementary figures and images for: Differential genetic responses to the stress revealed the mutation-order adaptive divergence between two sympatric ginger species
Source: BMC Genomics. 2018 Sep 21;19:692. doi: 10.1186/s12864-018-5081-3 (PMC6150995; doi:10.1186/s12864-018-5081-3)

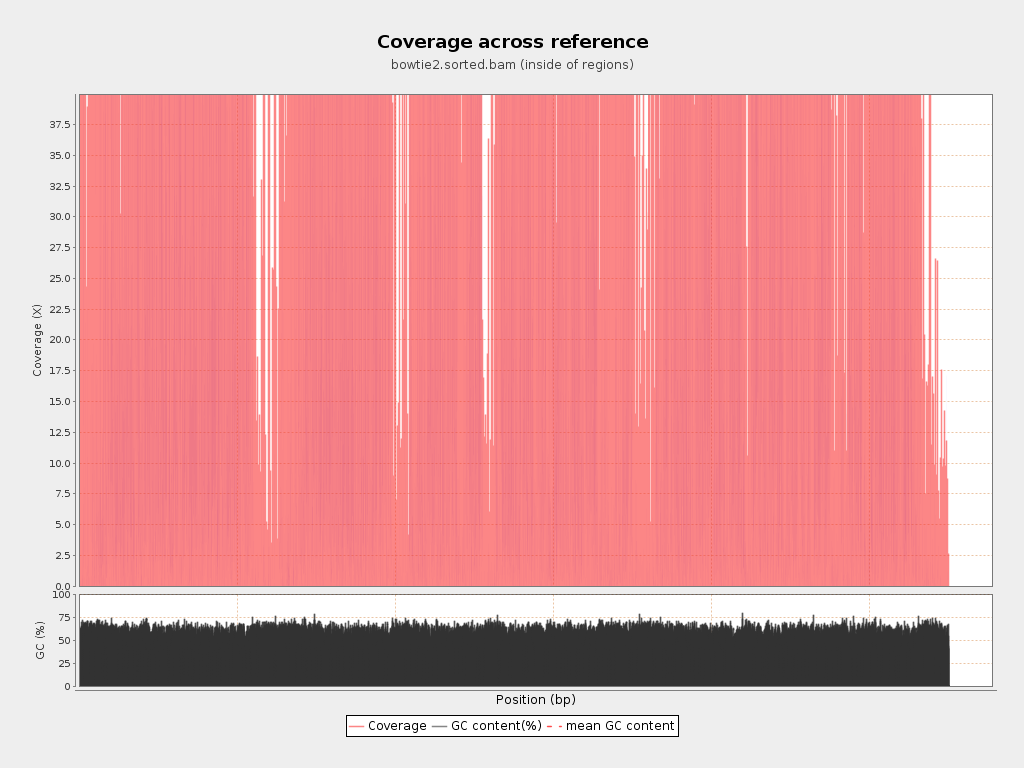

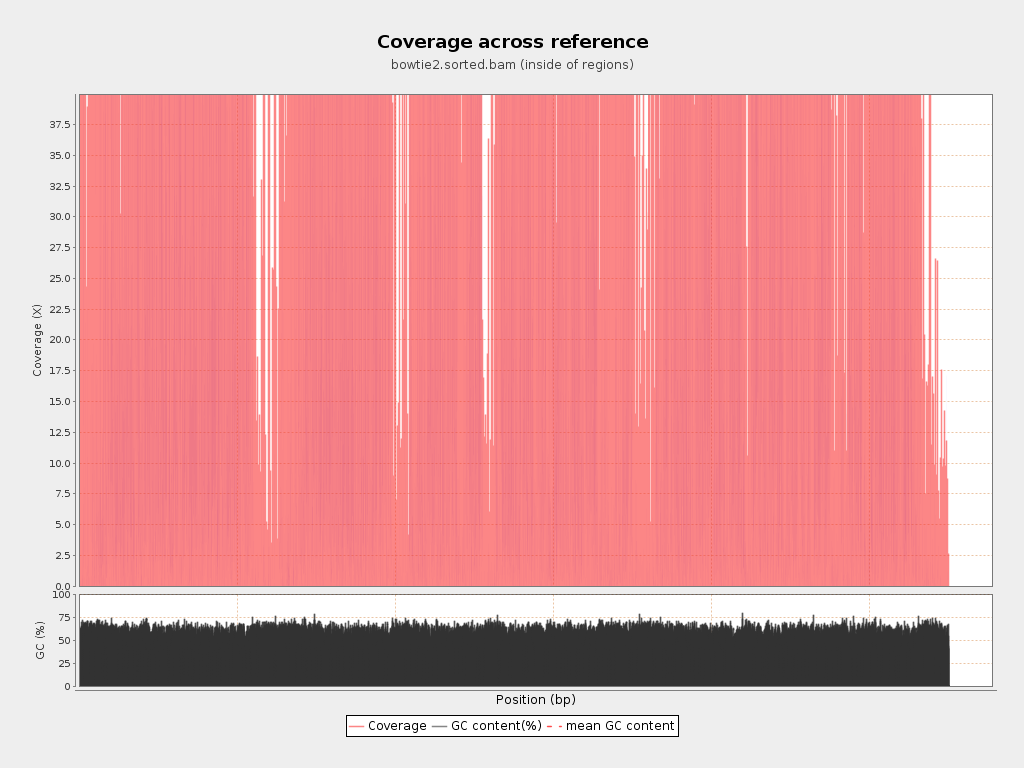


**Additional file 1** Sequencing depth of coverage across pseudo-reference genome.

Supplement: Supplementary file 1 — Sequencing depth of coverage across pseudo-reference genome. (DOCX 653 kb) [file 12864_2018_5081_MOESM1_ESM.docx]
